# The Synergistic Antitumor Effect of 5-Fluorouracil Combined with Allicin against Lung and Colorectal Carcinoma Cells

**DOI:** 10.3390/molecules25081947

**Published:** 2020-04-22

**Authors:** Adrian Bogdan Țigu, Vlad-Alexandru Toma, Augustin Cătălin Moț, Ancuța Jurj, Cristian Silviu Moldovan, Eva Fischer-Fodor, Ioana Berindan-Neagoe, Marcel Pârvu

**Affiliations:** 1 MedFuture Research Center for Advanced Medicine, University of Medicine and Pharmacy “Iuliu Hatieganu”, 400349 Cluj-Napoca, Romania; adrianbogdantigu@gmail.com (A.B.Ț.); moldovan.cristian1994@gmail.com (C.S.M.); fischer.eva@iocn.ro (E.F.-F.); ioana.neagoe@umfcluj.ro (I.B.-N.); 2 Faculty of Biology and Geology, Babeș-Bolyai University, 42 Republicii Street, 400015 Cluj-Napoca, Romania; vlad.al.toma@gmail.com; 3 Institute of Biological Research Cluj-Napoca, branch of NIRDBS Bucuresti, 400113 Cluj-Napoca, Romania; 4 Department of Molecular and Biomolecular Physics, National Institute for R&D of Isotopic and MolecularTechnologies, 67-103 Donat, 400293 Cluj-Napoca, Romania; 5 Department of Chemistry, Faculty of Chemistry and Chemical Engineering, Babes-Bolyai University,11 Arany Janos Street, 400028 Cluj-Napoca, Romania; gusty_chem@yahoo.com; 6 The Research Center for Functional Genomics, Biomedicine and Translational Medicine, “Iuliu Hatieganu” University of Medicine and Pharmacy, 400028 Cluj-Napoca, Romania; ancajurj15@gmail.com; 7 Department of Radiobiology and Tumor Biology, the Oncology Institute “Prof Dr Ion Chiricuta”, 400028 Cluj-Napoca, Romania; 8 Department of Functional Genomics and Experimental Pathology, the Oncology Institute “Prof Dr Ion Chiricuta”, 400028 Cluj-Napoca, Romania

**Keywords:** allicin, 5-FU, apoptosis, colony, synergistic effect, migration

## Abstract

5-fluorouracil (5-FU) is an anticancer drug used to inhibit the proliferation of many different tumor cells. Since severe events are associated with this compound, its combination with different anticancer drugs or adjuvants would allow the use of a significantly lower dose of 5-FU. In this study, we highlighted that the combination of allicin with 5-FU inhibited the cell migration and proliferation of colorectal and lung cancer cells. 5-FU inhibited cell growth with a similar inhibitory concentration for both normal and tumor cells (~200µM), while allicin showed different inhibitory concentrations. With an IC50 of 8.625 µM, lung cancer cells were the most sensitive to allicin. Compared to 5-FU and allicin single-agent treatments, the co-treatment showed a reduced viability rate, with *p* < 0.05. The morphological changes were visible on all three cell lines, indicating that the treatment inhibited the proliferation of both normal and tumor cells. We highlighted different cell death mechanisms—apoptosis for lung cancer and a non-apoptotic cell death for colorectal cancer. The synergistic antitumor effect of 5-FU combined with allicin was visible against lung and colorectal carcinoma cells. Better results were obtained when a lower concentration of 5-FU was combined with allicin than the single-agent treatment at IC50.

## 1. Introduction

Cancer is the second leading cause of death in the world and a major public health problem [1]. In 2018, around 1.73 million new cases of cancer were reported worldwide and there were more than 609,000 deaths in the United States alone [2]. According to Globocan, in 2018, 2 million new cases of lung cancer and more than 1.8 million new cases of colorectal cancer were reported. Both lung and colorectal cancers are estimated to be responsible for more than 2.5 million deaths worldwide [3].

Different synthesized chemical compounds are used for cancer therapy [4], and 5-fluorouracil (5-FU) is one of the most commonly used first-line anticancer drugs in clinical practice [5,6]. 5-FU is commonly prescribed for solid tumor types [7] and is widely used as an essential chemotherapeutic agent for colorectal cancer [6], gastric cancer [5], and hepatocellular carcinoma [8]. Moreover, 5-FU is most often used in combination with other specific cancer drugs to treat many types of cancer, including breast cancer, head and neck cancers, anal cancer, stomach cancer, colon cancer, and some skin cancers [9].

5-FU exerts its anti-cancer effect by preventing the synthesis of DNA and RNA in the cell by significantly decreasing thymidine [10]. The lack of functional DNA and RNA prevents the cancer cell from reproducing and making vital proteins, which leads to cell death. In this way, 5-FU slows or stops the growth of cancer cells in different oncologic conditions [11].

In addition, chemically synthesized anticancer agents can be extracted from plant sources [12], such as *Allium sativum* [8,13], *Curcuma longum* [1], and *Catharanthus roseus* [12,14], and these compounds can have different mechanisms of action. Furthermore, plant compounds such as allicin [8,15] from *Allium sativum*, curcumin [5] from *Curcuma longum* [1], and different alkaloids from *Catharanthus roseus* [12] are promising phytocompounds that can be used in the treatment of several types of cancer [13,14].

Allicin, an organosulfur compound that can be isolated from freshly crushed garlic (*Allium sativum* L.) or obtained by chemical synthesis [15,16], has been shown to possess numerous biological actions, such as anti-inflammatory and anti-microbial properties [16,17]. Additionally, several studies have reported that allicin represses cancer growth in vitro, including lung cancer, hepatocellular carcinoma, melanoma, and colorectal adenocarcinoma [18,19].

Since severe adverse events are associated with the anticancer treatment of 5-FU in clinical application [5], finding anticancer drugs from two or more compounds with different mechanistic actions which can enhance the cytotoxicity against tumor cells without having severe side effects on non-tumor cells is of great importance.

Different papers have reported the antitumor effects and molecular mechanisms of allicin in suppressing the malignant phenotype of cervical cancer cells, mainly by inhibiting the expression of NRF2 [20]; inhibiting proliferation and invasion in vitro and in vivo via SHP-1-mediated STAT3 signaling in cholangiocarcinoma [15]; and inducing apoptosis through the activation of both intrinsic and extrinsic pathways in glioma cells [21].

Previous research has reported that the anticancer effect of 5-FU is enhanced by different plant compounds, such as allicin [8] and curcumin [5].

Moreover, a synergistic anticancer effect was obtained by a combination of two plant extracts (artesunate from *Artemisia annua* and allicin from *Allium sativum*), which induced apoptosis in human osteosarcoma cells by increasing the activation of Caspase-3/9 [22].

The aim of our experiments was to explore if combinations of 5-FU and allicin have synergistic antitumor effects against two cellular lines represented by lung and colorectal carcinoma cells. Moreover, we investigated whether combinations with less 5-FU can produce similar or better results, by replacing this drug with synthesized allicin.

## 2. Results

### 2.1. The Effect of 5-FU and Allicin Alone and in Combination on the Proliferation of Colorectal Adenocarcinoma (DLD-1) and Lung Squamous Adenocarcinoma (SK-MES-1) Cells Analyzed by the 3-(4, 5-Dimethulthiazol-2-yl)-2,5-diphenyltetrazolium bromide (MTT) Assay

**Figure 1:**
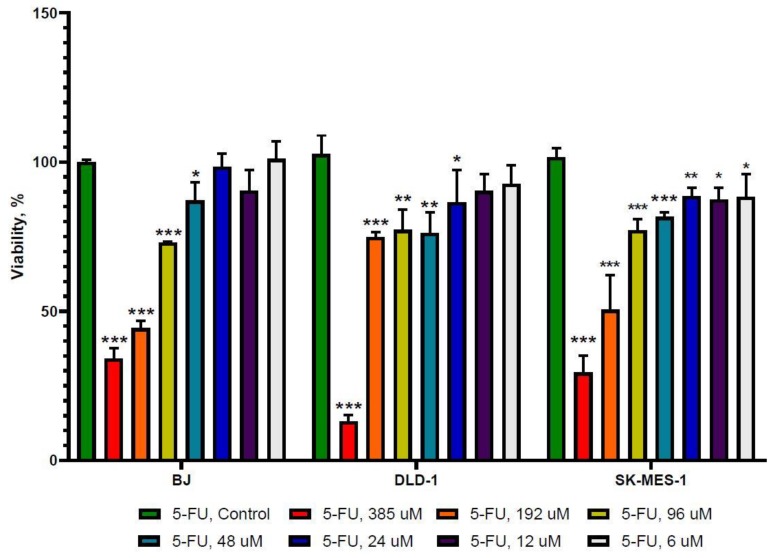
The viability rate analysis of 5-fluorouracil (5-FU) treatment. 5-FU inhibited cell growth in a dose-dependent manner in the case of normal fibroblasts (BJ), colorectal adenocarcinoma (DLD-1), and lung squamous adenocarcinoma (SK-MES-1) cells, with similar IC_50_, when incubated for 24 h (6, 12, 24, 48, 96, 192, and 385 μM 5-FU). The results with *p* < 0.05 were considered statistically significant (* *p* < 0.05, ** *p* < 0.01, and *** *p* < 0.001).

First, we evaluated the inhibitory effect of different concentrations of 5-FU and allicin alone and analyzed the cellular viability of normal fibroblasts (BJ), colorectal adenocarcinoma (DLD-1), and lung squamous adenocarcinoma (SK-MES-1) after 24 h treatment by the 3-(4, 5-dimethulthiazol-2-yl)-2,5-diphenyltetrazolium bromide (MTT) assay (Figure 1).

**Figure 2:**
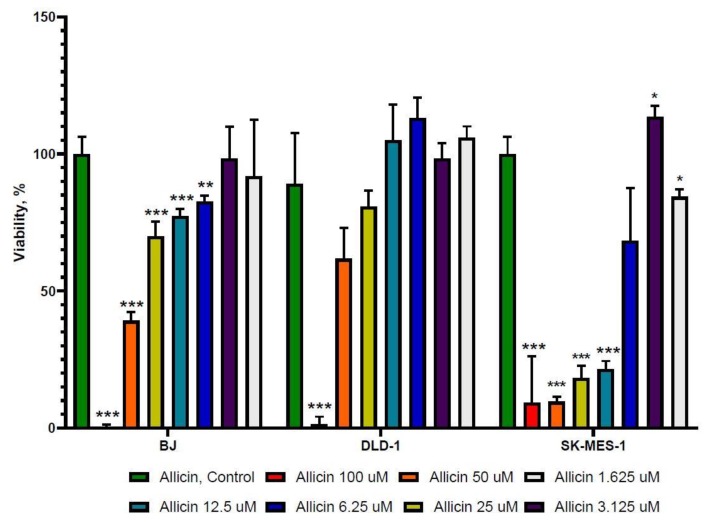
The viability rate analysis of allicin treatment. Allicin showed an inhibitory effect on BJ, DLD-1, and SK-MES-1 cells, with different IC_50_ for each cell line, when incubated for 24 h (1.625, 3.125, 6.25, 12.5, 25, 50, and 100 μM allicin). The results with *p* < 0.05 were considered statistically significant (* *p* < 0.05, ** *p* < 0.01, and *** *p* < 0.001).

As indicated in Figure 1, after 24 h of exposure to 5-FU, all three cell lines displayed growth inhibition at almost the same IC_50_, specifically, 195.9 μM for BJ, 214.3 μM for DLD-1, and 202.2 μM for SK-MES-1, indicating that the 5-FU effect is not specific to a certain cell type and inhibits the cell growth similarly for all cell lines. Allicin showed different IC_50_ values after 24 h treatment (Figure 2). The most sensitive cell line was SK-MES-1, with a value of 8.625 μM, followed by BJ cells, with a value of 33.17 μM, and the least sensitive cell line was DLD-1, with a value of 53.53 μM, showing different effects compared to 5-FU alone.

**Figure 3:**
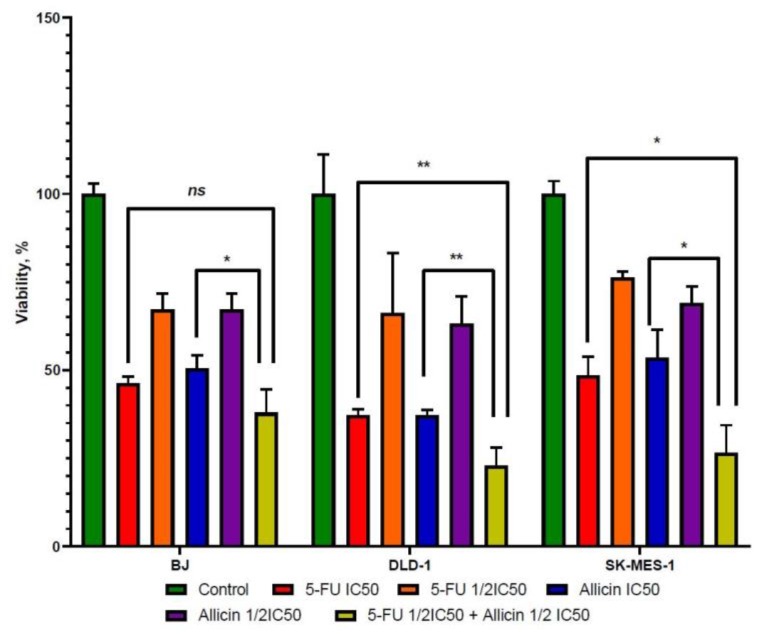
The viability rate analysis of 5-FU combined with allicin compared to each single-agent treatment IC_50_ dose. The comparison of the co-treatment and individual compounds indicated that the co-treatment was more effective against tumor cells compared to the cytotoxic drug and allicin alone. Abbreviations: NS, not significant; Control, untreated group; 5-FU IC50, group treated with 5-FU at IC_50_ dose; 5-FU 1/2 IC50, group treated with half of 5-FU IC_50_ dose; Allicin IC50, group treated with allicin IC_50_ dose; Allicin 1/2 IC50, group treated with half of allicin IC_50_ dose; 5-FU 1/2 IC50 + Allicin 1/2 IC50, group treated with the combination of 5-FU and allicin half IC_50_ doses. The results with *p* < 0.05 were considered statistically significant (* *p* < 0.05 and ** *p* < 0.01).

Second, we evaluated the combinatory effect of 5-FU and allicin. The antiproliferative effect of 5-FU and allicin combined at half IC_50_ concentrations was significantly greater than that of single-agent treatment, as presented in Figure 3. The antiproliferative effect on lung and colorectal cancer cells was enhanced when 5-FU was combined with allicin at half of their IC_50_, compared with 5-FU and allicin as single-agent treatment at IC_50_.

### 2.2. Establishment of Morphological Changes Induced after Single-Agent Treatment and Combined Treatment

**Figure 4:**
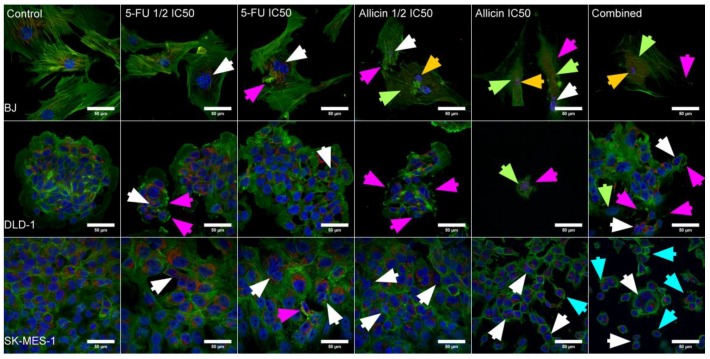
Confocal microscopy analysis of morphological changes induced by allicin, 5-FU, and co-treatment in BJ, DLD-1, and SK-MES-1 cells. The architecture of the cells is highlighted with Mitotracker-Red (mitochondrial networks, red), Phalloidin-FITC (cytoskeleton, green), and DAPI (nucleus, blue). The co-treatment showed a cytotoxic effect on all cell lines. The combination of allicin and 5-FU reduced the cell population in DLD-1 and SK-MES-1. Moreover, the cell shape was modified from normal to round, and gaps between cells were clearly visible (scale bar: 50 μm, 40 X objective). White arrows, nuclear damage; orange arrows, nuclear shrinkage; magenta arrows, cytoskeleton disruption; green arrows, mitochondrial activity decrease; cyan arrows, apoptotic volume decrease (AVD). BJ, normal fibroblasts; DLD-1, colorectal cancer cell line; SK-MES-1, lung cancer cell line; 5-FU ½ IC50, group treated with half of the 5-FU IC_50_; 5-FU IC50, group treated with 5-FU IC_50_; Allicin ½ IC50, group treated with half of the allicin IC_50_; Allicin IC50, group treated with Allicin IC_50_; combined, group treated with the combination of 5-FU and allicin at half IC_50_.

In order to evaluate the level of toxicity that was induced by the combined therapy and the morphological changes that were induced by the treatments, we used the triple staining protocol that was developed in our facility [23]. The mitochondrial networks were stained with Mitotracker-Red, the actin filaments were stained with Phalloidin-FITC, and the nucleus was stained with DAPI. As presented in Figure 4, the co-treatment induced cell death in all cell lines. In the case of the BJ cells, the morphological damage was largely attributed to the 5-FU, as this cell line has a much slower division rate when compared to the utilized tumor lines. Therefore, the fibroblasts were unable to regenerate and divide into new cells.

The colorectal cancer cell lines showed extensive damage and a decreased cell number associated with all treatment types, especially allicin. Specifically, allicin IC_50_ drastically reduced the cell numbers in the case of single treatment for all cell lines, while in the case of co-treatment, noticeable traits of apoptosis were also identified. Cells which were treated with half dosage, ½ IC_50_, for both compounds displayed morphological modifications attributed to cellular stress.

The lung cancer cells showed nuclear damage, mostly induced by allicin, and the cells that were under combined treatment showed an apoptotic volume decrease (AVD); this feature was only present in the lung cancer cells after co-treatment and few cells presented AVD after allicin treatment. On the other hand, mostly in BJ cells, a mitochondrial activity decrease was observed after allicin and co-treatment, and nuclear shrinkage was also attributed to this cell line. In the case of the DLD-1 cell line, the cytoskeleton disruption was more intense compared to the other cells, but the other apoptotic specific features were less visible.

Taken together, these results indicate that 5-FU and allicin co-treatment induced morphological changes in the case of colorectal cancer cells, including cytoskeleton disruption, nuclear damage, and reduced mitochondrial activity, while in the case of SK-MES-1, the apoptotic volume decrease was the main feature, which was attributed to this cell line after co-treatment, indicating cell death induced by apoptosis.

In the images depicting the SK-MES-1, we observed that 5-FU inhibited cell division and growth, as there are noticeable gaps between the cells, and the allicin-treated cells display changes in their architecture, in the form of becoming more spherical and acquiring apoptotic-specific traits, such as nucleus breakage and AVD. These results indicate that 5-FU, allicin, and co-treatment induced visible morphological changes in DLD-1 and SK-MES-1 cells.

### 2.3. In Vitro Co-Treatment Inhibited Cell Migration and Colony Formation

**Figure 5:**
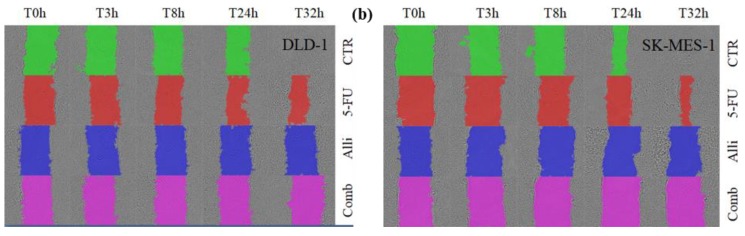
The investigation of the migration and colony formation capacity after allicin, 5-FU, and co-treatment. (**a**) DLD-1 cells are sensitive to allicin and co-treatment, while the 5-FU has a limited effect on cell migration. (**b**) SK-MES-1 cells are sensitive to combined treatment and allicin, with 5-FU being unable to totally inhibit migration. Abbreviations: CTR, control group; 5-FU, half of the 5-fluorouracil IC_50_-treated group; Alli, half of the Allicin IC_50_-treated group; Comb, the co-treatment group.

Cell invasion and migration are key processes during tumor formation and growth; thus, we evaluated the inhibitory effect of our compounds on cell migration and colony formation. The inhibition of these mechanisms can stop tumor cell migration and the development of metastatic sites. We examined whether synthesized allicin and 5-FU combined treatment can inhibit cellular migration processes, as well as colony formation and development (Figure 5).

**Figure 6:**
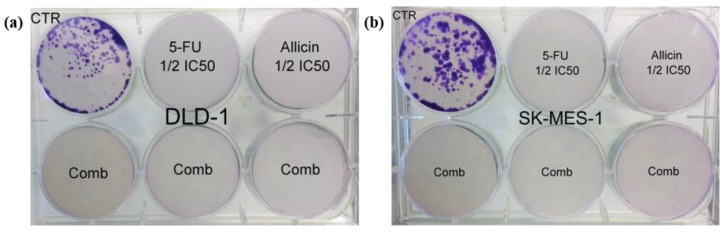
The investigation of the migration and colony formation capacity after allicin, 5-FU, and co-treatment. The DLD-1 (**a**) and SK-MES-1 (**b**) colony formation was inhibited by single-agent treatments and co-treatment. Abbreviations: CTR, control group; 5-FU 1/2IC_50_, half of the 5-fluorouracil IC_50_-treated group; Allicin 1/2 IC_50_, half of the Allicin IC_50_-treated group; Comb, the co-treatment group.

Cell migration was inhibited by all treatments, with the combined treatment being the most effective against cell migration, while the 5-FU as a single-agent treatment was the least effective against cell migration in both tumor cell lines. Colony formation was inhibited by all treatments, indicating that the cells’ ability to divide was inhibited by 5-FU, allicin, and co-treatment (Figure 6).

All these results indicated that the combined treatment reduced migration, mostly due to allicin, and colony formation was inhibited.

### 2.4. The Evaluation of Apoptosis and Necrosis after Single-Agent Treatments and Co-Treatment In Vitro Administration

**Figure 7:**
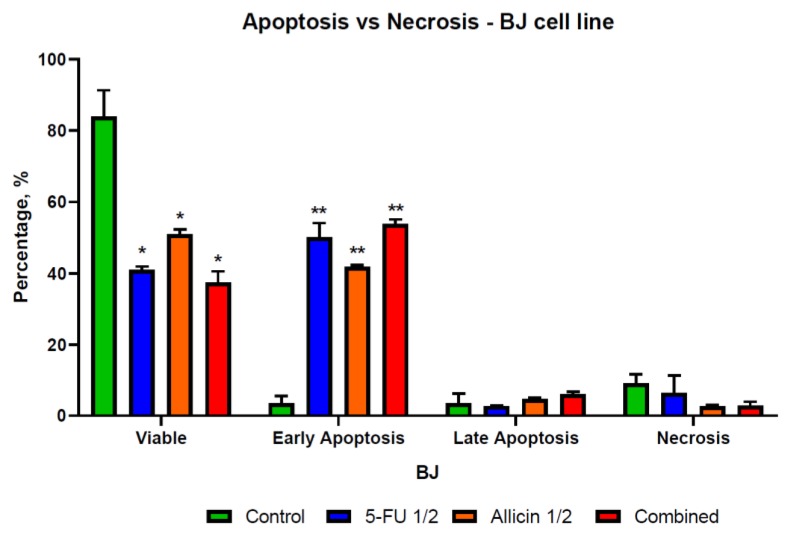
The illustration of apoptosis and necrosis in the BJ cell population. BJ cell line ratio between viable, apoptotic, and necrotic cells, with an increased number of early apoptotic cells, for all treatments. Abbreviations: Control, control group; 5-FU 1/2, half of the 5-FU IC_50_-treated group; Allicin 1/2, half of the Allicin IC_50_-treated group; Combined, the co-treatment group. The results with *p* < 0.05 were considered statistically significant (* *p* < 0.05 and ** *p* < 0.01).

**Figure 8:**
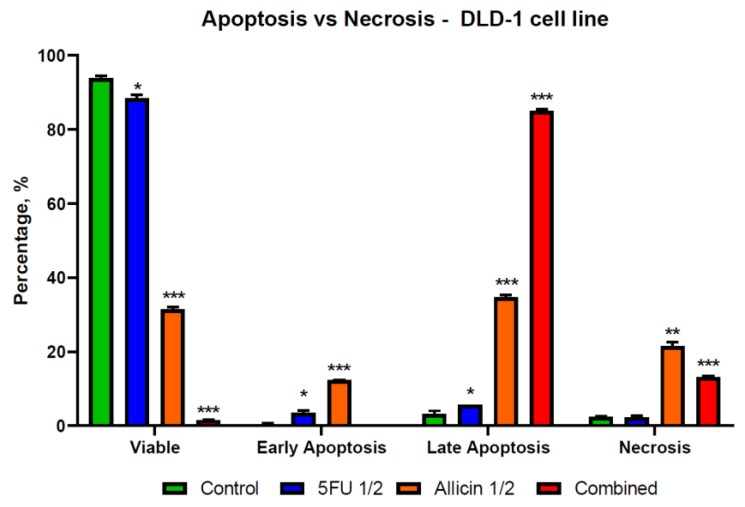
The illustration of apoptosis and necrosis in the DLD-1 cell population. DLD-1 cell line ratio between viable, apoptotic, and necrotic cells, with an increased number of late apoptotic cells for combined treatment. Abbreviations: Control, control group; 5-FU 1/2, half of the 5-FU IC_50_-treated group; Allicin 1/2, half of the Allicin IC_50_-treated group; Combined, the co-treatment group. The results with *p* < 0.05 were considered statistically significant (* *p* < 0.05, ** *p* < 0.01, and *** *p* < 0.001).

**Figure 9:**
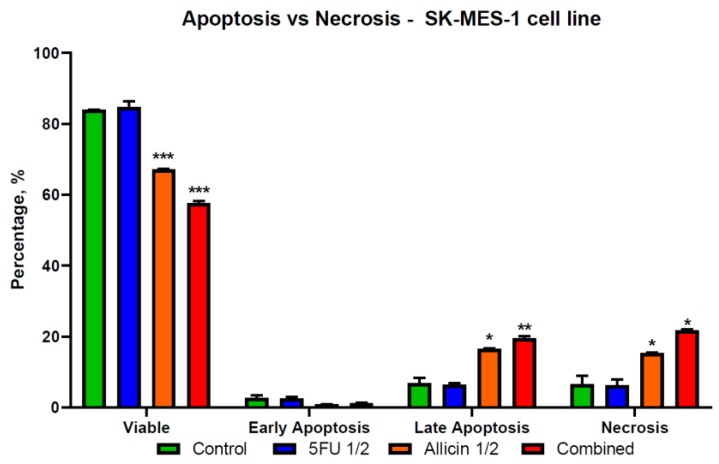
The illustration of apoptosis and necrosis in the SK-MES-1 cell population. SK-MES-1 cell line ratio between viable, apoptotic, and necrotic cells, with an increased number of late apoptotic and necrotic cells. The graphics were analyzed using Prism8 software. Abbreviations: Control, control group; 5-FU 1/2, half of the 5-FU IC_50_-treated group; Allicin 1/2, half of the Allicin IC_50_-treated group; Combined, the co-treatment group. The results with *p* < 0.05 were considered statistically significant (* *p* < 0.05, ** *p* < 0.01, and *** *p* < 0.001).

We examined the rate of apoptosis and necrosis using the Annexin V/P.I. flow cytometry assay. The results indicate that the allicin and 5-FU act as stimulators of early apoptosis in normal cells (Figure 7), while the DLD-1 and SK-MES-1 cell lines showed more of a late apoptotic distribution shifting to necrosis (Figure 8 and Figure 9). The co-treatment was the most effective in inducing cell death, mostly on DLD-1 cells, while on SK-MES-1 cells (Figure 9), the ratio of viable cells to apoptotic cells was higher compared to DLD-1 (Figure 8). A possible explanation for this is related to the cell morphology; due to their round shape, many SK-MES-1 cells were detached and were not captured when the sample was processed or were detected as debris.

**Figure 10:**
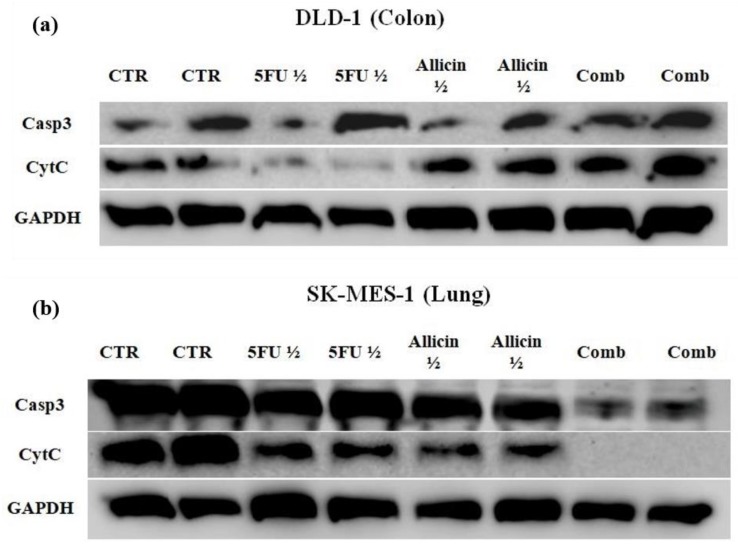
The western blot analysis of Caspase 3 and Cytochrome C in colorectal and lung cancer cells. (**a**) Western blot on DLD-1 cells, where GAPDH was used as the control and Caspase 3 and Cytochrome C were determined. (**b**) Western blot on SK-MES-1 cells, where the same molecules were determined as for DLD-1. Abbreviations: GAPDH, Glyceraldehyde 3-phosphate dehydrogenase; CytC, Cytochrome C; Casp3, Caspase 3; CTR, control; 5-FU half of the IC_50_, 5FU ½; allicin half of the IC_50_, Allicin ½; and “comb”, combination of the half IC_50_.

Apoptosis was further evaluated by immunoblotting, which was conducted to investigate the main components of both intrinsic and extrinsic apoptotic pathways. The levels of Caspase 3 and Cytochrome C were evaluated, while Glyceraldehyde 3-phosphate dehydrogenase (GAPDH) was used for normalization. In the case of the DLD-1 cell line, there were no noticeable differences between the bands corresponding to Caspase 3. On the other hand, Cytochrome C was decreased after 5-FU treatment, while after allicin and combined treatment, the level of Cytochrome C was the same as the control or a bit higher (Figure 10a).

The results indicated that 5-FU triggered apoptosis, while allicin and co-treatment may act in a different manner, in order to induce cell death. The Cytochrome C that was detected by western blot analysis is the free total Cytochrome C, which is not part of the apoptosome complex, due to the fact that we used whole cellular homogenate, without splitting it into cytosolic and mitochondrial fractions by ultracentrifugation. For SK-MES-1, the result confirmed that the combined treatment induced apoptosis, total Caspase 3 was significantly reduced, and free Cytochrome C was not detected (Figure 10b).

The combined treatment has different effects in a cell-dependent manner; in both cases, cell death was induced.

## 3. Discussion

Lung cancer is an important cause of death in developed countries [24]; lung adenocarcinoma accounts for almost 50% of all lung cancers [25]. Due to its aggressiveness and late diagnosis, therapy against lung adenocarcinoma is not efficient all the time [26]. Colorectal cancer (CRC), the third most common type of cancer worldwide, has a high incidence in western countries; the risk of CRC development is dependent on lifestyle [27]. Both types of cancer cause a significant number of deaths every year [3]. Environmental factors, lifestyle, a lack of screenings, and late therapeutic intervention can result in late stage of cancer development, and all can hinder the overall therapeutic efficiency. The aim of the scientific world is to develop therapies that eliminate cancer cells, whilst having minimal effects on normal tissues and cells. Surgery, radiation, and chemotherapy are used to fight cancer, but with a limited efficiency, and in some cases, tumors become resistant to all therapy regimens or the therapy is too aggressive for normal tissues [28]. In this context, alternative treatments are needed to enhance the conventional therapies.

5-FU has been used in the last decades to improve the survival of patients diagnosed with cancer. Its mechanism of action is focused on the interaction with nucleic acids producing DNA damage by mismatch repair deficiency [29], which inhibits DNA synthesis, and in combination with other therapies, it can enhance the anti-proliferative effects on lung cancer [30]. Moreover, 5-FU is one of the most commonly used antitumor drugs against colorectal cancer, with some limitations due to the resistance to therapy [31].

One strategy for fighting against drug resistance and drug-induced toxicity is to add adjuvant treatments in combination with classic therapy, in order to reduce the toxic dose of chemotherapy and to replace it with other agents, with a natural origin, that are less aggressive against normal tissues, when the compounds are standardized and stabilized. In this regard, our study is based on the synergistic effect of the in-house-synthesized allicin, as an adjuvant treatment in combination with 5-FU, which is a cytotoxic compound widely used in cancer treatment. Natural compounds interact with different molecular mechanisms and depending on the disease or therapeutic molecules, some bioactive compounds can reduce DNA damage and oxidative stress, can inhibit proliferation, and can reduce genetic and proteomic alterations, and can thus be used as antitumor agents [19,32,33,34,35].

In this study, we used allicin, which is a natural compound with high potential as an adjuvant agent. The main active compound isolated from *Allium sativum* has many biological effects, which have been demonstrated during the last decades. Its antioxidant, anti-inflammatory, immunostimulatory, and antifungal activities have been the study subjects for many research groups [16,36]. A constant intake of allicin via garlic consumption could reduce chronic inflammation, oxidative stress, and DNA damage [37,38,39].

In this study, the acute effect of 24h exposure to single-agent treatment and co-treatment was investigated, based on the 24h IC_50_, in accordance with other research papers that have previously been published by other research groups [40,41]. Allicin 24h treatment was shown to be efficient in lung, breast, and colon cancer [42], and thyroid cancer [43]. Allicin can inhibit cell the migration and proliferation of lung adenocarcinoma after 24h treatment [40,44]. Furthermore, the 24h co-treatment of allicin combined with 5-FU showed that allicin sensitizes hepatocarcinoma cells to 5-FU, stimulating drug uptake [8].

In the present study, allicin inhibited migration and promoted cell death in the investigated lung and colorectal tumor cell lines [40,44]. The biological effects of allicin are attributed to the thiol groups. The active organosulfur compound was potent in disrupting the microtubules by interfering with tubulin polymerization. By inhibiting the microtubule polymerization, allicin inhibited cell polarization and migration, and reduced cell division [45,46]. Its antiproliferative activity was increased in a dose-dependent manner against human cervical cancer cells [20], cholangiocarcinoma [15], lung adenocarcinoma [44] renal cell carcinoma [47], and ovarian cancer cells [48]. Furthermore, allicin acts as a good inhibitor of colony formation, as well as cell migration [15]. As is demonstrated in the consulted literature, the synthesized allicin that we used in this study inhibited cell migration and colony formation. In combination with 5-FU, the inhibitory effect was more pronounced, and the most visible changes were observed for the morphological details of the cytoskeleton and the cell shape, indicating the triggering of cell death. The most visible morphological changes were observed in SK-MES-1 cells, and the apoptotic volume decrease was specific for lung cancer cells after co-treatment. Moreover, the nuclear damage confirmed the initiation of apoptosis. The apoptotic volume decrease was reported to be a sign of cell death that occurs through apoptosis [49].

Cell death can be induced by either programmed cell death like apoptosis and autophagy, or necrosis [50]. We also investigated this aspect and were able to highlight different cell death mechanisms induced by 5-FU and allicin, depending on the cell lines used. Apoptosis is a mechanism that can be activated by oxidative stress induced by xenobiotics exposure [51] or by pro-apoptotic therapeutic agents, such as cytostatic drugs or adjuvant compounds. Both extrinsic and intrinsic pathways converge into Caspase 3 (Casp3), which is the intrinsic pathway modulated by the mitochondrial Cytochrome C (CytC). Intracellular stress activates the mitochondrial pathway, where the proapoptotic molecules Bid (BH3 interacting-domain death agonist), Bak (Bcl-2 homologous antagonist/killer), and Bax (Bcl-2-associated X protein) promote the release of CytC that further binds to the apoptosome, which activates Caspase 9. This cascade of events can finally activate Casp3. On the other hand, in the case of extrinsic apoptosis, Caspase 8 is activated by signaling through death receptors and it can further activate Casp3, which is the common player in both apoptotic pathways. Moreover, Caspase 8 can cleave Bid and this event will further activate the mechanism of the intrinsic pathway [52,53].

Apoptosis occurs via the activation of caspases, and cysteine proteases that are the effectors of apoptosis. Casp3 is the common player of both extrinsic and intrinsic apoptosis, and is activated by CytC release or the activation of Caspase 8 [54]. Mitochondria harbor the activation cascade of apoptosis, reactive oxygen species (ROS) are produced, the membrane potential is changed, and CytC is released. Moreover, the apoptosis inducing factor (AIF) is released, together with CytC [54]. We observed a lower concentration of the total Casp3 in the SK-MES-1 after co-treatment compared with single-agent treatments, implying the synergistic effect of 5-FU and our synthesized allicin. We showed that the cytotoxic effect induced by the treatments could be correlated with a reduced signal of CytC in the western blot assay, meaning that the more efficient the treatment was, the lower concentration of free CytC was, due to its integration in the apoptosome complex.

When CytC is released and becomes part of the apoptosome complex, it will activate Caspase 9, which will further activate pro-Caspase 3 that will be cleaved and become active, therefore inducing cell death. Extrinsic apoptosis exhibits crosstalk with the intrinsic pathway via Bid, which is activated by Caspase 8, further promoting CytC release [55]. The chemotherapeutic agents usually activate intrinsic apoptosis, while extrinsic apoptosis is activated by antibodies or extracellular ligands like TRAIL [56].

Other researchers have demonstrated that allicin induces redox toxicity and is able to activate apoptosis via a redox-mechanism [42,57]. In our study, apoptosis was induced in the SK-MES-1 cells, where the amount of Casp3 that was not cleaved was significantly lower in the co-treatment group compared to the single-agent treatments. On the other hand, in the case of DLD-1 cells, a non-apoptotic type of cell death, probably necrosis, or a programmed cell death that is caspase-independent, was activated. In the CRC, the Casp3 levels were not different between the experimental groups. The antitumoral effect was visible after 24h single-agent treatment and co-treatment, similar to other research papers that have previously been published [40,41,43,44] The main cancer features, such as cell migration and colony formation, were inhibited by the short-term treatment. Moreover, in the case of lung cancer cells, apoptosis was initiated.

The results suggest a late apoptosis state for both tumor cell lines included in this study, compared to normal cells that underwent early apoptosis, indicating that the toxicity was induced with a delay in normal cells compared to the tumor cells. It is essential to investigate the efficacy of co-treatments and single-agent treatments against other CRC and lung cancer cell lines and to further investigate the antitumor effects on in-vivo models.

Our results are in accordance with other papers and proved that allicin has anticancer potential [12,13,15] against two different cellular lines and that 5-FU combined with allicin has a synergistic antitumor effect on hepatocellular cancer cells [8].

## 4. Materials and Methods

### 4.1. Cell Culture Reagents

BJ (ATCC^®^ CRL-2522^™^) normal fibroblasts, SK-MES-1 (ATCC^®^ HTB-58^™^) squamous lung cells, and DLD-1 (ATCC^®^ CCL-221^™^) colorectal carcinoma cells were acquired from American Type Culture Collection (ATCC, Manassas, VA, USA) and were maintained in Roswell Park Memorial Institute (RPMI) cell culture medium, supplemented with 10% Fetal Bovine Serum, 1% Glutamine, and 1% Penicillin. All the reagents were purchased from Gibco (Grand Island, NY, USA). During the experiment, the cells were stored in a humidified incubator at 37°C and 5% CO_2_.

5-FU was purchased from Sigma Aldrich (Sigma-Aldrich, St. Louis, MO, USA) and the working concentrations were calculated according to the stock concentration.

### 4.2. Allicin Synthesis and Characterization

Allicin was synthesized and characterized as reported in our previous work, and using the Mass Spectrometry (MS) and ^1^H, and ^13^C NMR analysis, we determined that allicin has a purity of more than 96% [16].

### 4.3. Cell Viability Assay

The effects of allicin and 5-FU treatments were investigated using the 3-(4, 5-dimethulthiazol-2-yl)-2,5-diphenyltetrazolium bromide (MTT) assay. Cells were grown at a density of 1 × 10^4^ cells/well in a 96-well flat-bottom plate. After 24 h incubation, cells were treated with allicin and 5-FU and incubated for one day at 37°C in a humidified incubator with 5% CO_2_ [8]. The absorbance at 570 nm, corresponding to the viability rate of the cells, was measured using the MTT assay (Sigma-Aldrich, St. Louis, MO, USA). The plates were analyzed with TECAN SPARK10M (TECAN, Austria GmbH, Grodig). The viability rate and IC_50_ values were calculated using Prism 8 software (https://www.graphpad.com/guides/prism/7/user-guide/citing_graphpad_prism.htm).

### 4.4. Combined Treatment’s Effect on Cell Viability

The effect of combined half IC_50_ of allicin and 5-FU was investigated using the MTT assay. Cells were grown at the same density as in the cell viability assay. After one day of incubation, cells were treated with the IC_50_ concentrations, half of these doses, and the combined treatment of both half of the IC_50_ doses for 24 h [8]. The absorbance of the MTT was measured at 570 nm, with TECAN SPARK10 M (TECAN, Austria GmbH, Grodig). The viability rate was calculated using Prism 8 software.

### 4.5. Morphological Analysis of Confocal Microscopy

After 24 h of exposure to the half of the IC_50_ and combined IC_50_ treatments, the morphological changes induced by the treatments were assessed using the triple staining protocol previously published by our research team [23]. The cells were incubated on a chamberslide for 24 h with the treatment added. After 24 h, the cells were washed and stained with Mitotracker (Thermo Fisher Scientific, Waltham, MS, USA) for labeling the mitochondria (Texas Red), Phalloidin-FITC (Cytoskeleton, Denver, CO, USA) for labeling the actin filaments, and DAPI (Thermo Fisher Scientific, Waltham, MS, USA) for labeling the nucleus. Cells were analyzed under an Olympus FLUOVIEW FV1200 laser scanning fluorescence confocal microscope (Olympus, Tokyo, Japan).

### 4.6. Apoptosis and Necrosis Assessment by Flow Cytometry Analysis

In order to detect the apoptosis and necrosis ratio after 24 h treatment, the cells were washed with cold PBS and the pellet was resuspended in 100 μL of Annexin V Binding buffer. After a gentile mixing step, 5 μL of Annexin V and 5 μL of propidium iodide were added and each sample was incubated for 15 min in the dark at room temperature [23]. All the reagents were purchased from Thermo Fisher Scientific, Waltham, MS, USA. Afterwards, the samples were centrifuged and resuspended in 500 μL of cell wash solution and analyzed using a BD FACS Flow cytometer (BD, San Jose, CA, USA). The graphics were analyzed using Prism8 software.

### 4.7. Wound Healing Assay

Colorectal and lung cancer cells were seeded in 6-well flat-bottom plates. A total of 1 × 10^5^ cells were added to each well and incubated overnight at 37 °C. After one day, the cells were treated for 24 h and after the treatment, the cell culture medium was changed with free Fetal Bovine Serum (FBS) cell culture medium. The scratch was made using a pipette tip and the wound healing was monitored until the wound of the control group was closed [58]. The images were processed using ImageJ software (National Institutes of Health, Bethesda, MD, USA).

### 4.8. Colony Assay

For each treatment and control, for both cell lines, 500 cells were seeded in a 6-well plate (Eppendorf, Hamburg, Germany). After 24 h treatment, the cell culture medium was changed with treatment-free cell culture medium and the cells were analyzed each day to evaluate the colony formation. After two weeks, the colonies were analyzed by fixing the sample with pure methanol (Sigma-Aldrich, St. Louis, MO, USA) and staining the cells with violet crystals (Sigma-Aldrich, St. Louis, MO, USA) [59].

### 4.9. Western Blot

Cells were resuspended in Trizol and the protein fraction was further washed with 96% ethanol. The proteins were precipitated with 2-propanol and the pellet was washed three times with 0.3 M Guanidine-HCl in 96% ethanol. The pellet was resuspended in 1:1 solution of 1% SDS:8 M Urea in 1 M Tris-HCl. All the reagents for protein extraction were purchased from Sigma Aldrich, St. Louis, USA. After five cycles of 15 s of sonication steps, the samples were stored at −80°C. Equal amounts of proteins, quantified using the Nanodrop 2000c system (Thermo Fisher Scientific, Waltham, MS, USA), were separated in precast Mini-Protean TGX gels (Bio-Rad, Hercules, CA, USA) and transferred onto 0.45 μm nitrocellulose membranes (Bio-Rad, Hercules, CA, USA). After blocking with 5% nonfat dry milk in Tris-buffer saline with Tween 20 (Invitrogen, Carlsbad, CA, USA) (TBST), the membranes were incubated for 2 h at room temperature with a primary antibody for Caspase-3 (Abcam, Cambridge, UK), Cytochrome C (Abcam, Cambridge, UK), and GAPDH (Abcam, Cambridge, UK), with stripping steps between primary antibody incubations. After washing with TBST, the membranes were incubated with secondary antibodies conjugated with horseradish peroxidase. The signals were detected after adding the chemiluminescence substrate (Invitrogen, Carlsbad, CA, USA) [8,20].

### 4.10. Statistical Analysis

All the results were expressed as the mean ± standard deviation (SD). The statistical analysis was performed using Prism8 software by T-student. The calculations were made in comparison with the control group or treated groups. A difference at *p* < 0.05 was considered statistically significant (* *p* < 0.05, ** *p* < 0.01, and *** *p* < 0.001).

## 5. Conclusions

Synthesized allicin is a potent antitumor agent and due to its beneficial health effects, the co-treatment of allicin with 5-FU can be employed in multiple therapeutic approaches inhibiting migration and colony formation, which are two main features of tumor cells. By inhibiting migration and colony formation, the co-treatment prevents tumor cells from spreading and creating secondary sites.

The cell death mechanism induced by our synthesized allicin requires further investigations, and analysis of the cross talk between apoptosis, necrosis, redox biological machinery, and other key biological processes can reveal the deep and complex mechanism of cell death induced by synthesized allicin.

This paper shows, for the first time, that 5-FU combined with allicin has a synergistic antitumor effect against two kinds of tumor cell lines represented by lung and colorectal carcinoma cells. These results complete literature data regarding the synergistic antitumor effect of the combination against tumor cell lines. In future work, the aim will be to investigate the effects of 5-FU combined with allicin against other cancer types by conducting in vivo research, which is required to evaluate the toxicity of allicin and 5-FU and develop new strategies for a better targeted delivery system.
